# Hmo1 Protein Affects the Nucleosome Structure and Supports the Nucleosome Reorganization Activity of Yeast FACT

**DOI:** 10.3390/cells11192931

**Published:** 2022-09-20

**Authors:** Daria K. Malinina, Anastasiia L. Sivkina, Anna N. Korovina, Laura L. McCullough, Tim Formosa, Mikhail P. Kirpichnikov, Vasily M. Studitsky, Alexey V. Feofanov

**Affiliations:** 1 Biology Faculty, Lomonosov Moscow State University, 119992 Moscow, Russia; 2 Department of Biochemistry, University of Utah School of Medicine, Salt Lake City, UT 84132, USA; 3 Shemyakin-Ovchinnikov Institute of Bioorganic Chemistry, Russian Academy of Sciences, 117997 Moscow, Russia; 4 Fox Chase Cancer Center, Philadelphia, PA 19111, USA

**Keywords:** nucleosome, Hmo1, FACT, high mobility group B protein, H1 histone, spFRET, EMSA

## Abstract

Yeast Hmo1 is a high mobility group B (HMGB) protein that participates in the transcription of ribosomal protein genes and rDNA, and also stimulates the activities of some ATP-dependent remodelers. Hmo1 binds both DNA and nucleosomes and has been proposed to be a functional yeast analog of mammalian linker histones. We used EMSA and single particle Förster resonance energy transfer (spFRET) microscopy to characterize the effects of Hmo1 on nucleosomes alone and with the histone chaperone FACT. Hmo1 induced a significant increase in the distance between the DNA gyres across the nucleosomal core, and also caused the separation of linker segments. This was opposite to the effect of the linker histone H1, which enhanced the proximity of linkers. Similar to Nhp6, another HMGB factor, Hmo1, was able to support large-scale, ATP-independent, reversible unfolding of nucleosomes by FACT in the spFRET assay and partially support FACT function in vivo. However, unlike Hmo1, Nhp6 alone does not affect nucleosome structure. These results suggest physiological roles for Hmo1 that are distinct from Nhp6 and possibly from other HMGB factors and linker histones, such as H1.

## 1. Introduction

The initial level of chromatin compaction results from the assembly of nucleosomes—supramolecular complexes formed by wrapping DNA ~1.7 times around histone octamers [1]. The formation of nucleosomes effectively compacts the DNA and also creates a barrier to the accessibility of various factors involved in DNA transcription, replication, and repair. Therefore, regulating these vital cellular processes involves modulating chromatin structure through the actions of various architectural proteins, remodeling complexes, and histone-modifying enzymes.

High mobility group B (HMGB) factors constitute a family of non-histone architectural proteins that contain a distinct DNA-binding domain. In the yeast *Saccharomyces cerevisiae,* this family is represented by Nhp6A, Nhp6B, Nhp10, Hmo1, Abf2, Ixr1, and Rox1 [2]. Together, these HMGB proteins are highly abundant in the yeast nucleus (~1 molecule per 1–2 nucleosomes) [3,4], and they participate in chromatin remodeling, regulation of transcription, and DNA repair (see [5,6] for review).

The two Nhp6 paralogs (Nhp6A/B [7]) are the most abundant HMGB proteins in yeast cells with about 14,000 and 8000 molecules per cell, respectively [3]. Nhp6 is involved in regulating RNA polymerase II and III transcription [8,9,10], and interacts with several remodeling complexes including Swi/Snf (SWItch/Sucrose Non-Fermentable) [11] and RSC (Remodeling the Structure of Chromatin) [12].

Nhp6 is also required for the ATP-independent, reversible unfolding of nucleosomes by FACT (FAcilitates Chromatin Transcription) in vitro [13,14,15]. FACT is a highly conserved histone chaperone with many functional roles [16,17,18,19,20,21], but unlike the Spt16-SSRP1 composition found in most eukaryotes, yeast FACT is an Spt16-Pob3 heterodimer that lacks the HMGB domain found in SSRP1 [22,23,24,25]. The role of this DNA-binding domain in FACT activity and the reasons for the variable architecture among organisms remain only partially understood, but Nhp6 appears to support FACT activity both in vitro and in vivo [22,23,26]. However, while Spt16 and Pob3 are both essential for the viability of *S. cerevisiae*, cells lacking Nhp6 display a severe growth defect but remain viable. It has therefore been proposed that other factors might partially support FACT activity [7,22,26].

One of the candidates for this role is Hmo1, a highly abundant yeast HMGB protein (~13,000 molecules per cell; 1 molecule per 4–5 nucleosomes) [3]. Hmo1 is associated with promoters of ribosomal protein genes and directly participates in the transcription of these genes and of the rDNA locus [27,28,29]. Purified Hmo1 protein promotes binding of Swi/Snf complexes to nucleosomes, transfer of histone octamers, and exposure of nucleosomal DNA during chromatin remodeling [11]. It also enhances the activities of the RSC and ISW1a remodelers [28,30]. It is therefore likely that Hmo1 alters a structural feature of nucleosomes that supports remodeling in a general way.

Like mammalian HMGB1, Hmo1 has two HMGB DNA-binding domains, but it also has a C-terminal region enriched in basic amino acids not found in other HMGB proteins [31]. Hmo1 binds to DNA in a sequence non-specific manner and has increased affinity for non-canonical DNA structures like four-way junctions and supercoils [32]. Like Nhp6, Hmo1 bends DNA [33,34,35]. It has been suggested that Hmo1 might bind specifically to nucleosomal linker DNA [28,36], and like linker histones, play a role in stabilizing chromatin [37,38].

Here we report that Hmo1 affected the structure of nucleosomes and chromatosomes and supported the nucleosome-unfolding activity of FACT. Hmo1 binding was not limited to nucleosomal linker DNA, but instead also occurred in the core region, resulting in a change in the conformation of both nucleosomal and linker DNA that was distinct from the effects of the linker histone H1. Hmo1 also facilitated the binding of FACT to nucleosomes, enhanced its ability to promote ATP-independent, reversible unfolding of nucleosomal DNA, and partially supported FACT function in vivo. These results suggest physiological roles for Hmo1 that are distinct from those of other HMGB factors and linker histones. 

## 2. Materials and Methods

### 2.1. Reagents

Taq DNA polymerase, dNTP, and 10× Taq buffer were obtained from Evrogene (Moscow, Russia).

### 2.2. Protein Expression

Nhp6 was expressed in *Escherichia coli* and purified as described [39,40]. Yeast FACT was purified as a heterodimer (Spt16/Pob3) from yeast cells overexpressing both proteins [41,42].

The *Saccharomyces cerevisiae HMO1* ORF was amplified from genomic DNA as a 784 NdeI-BamHI fragment with primers pTF1608 and pTF1609, and inserted into a modified version of pET11A, resulting in an 8X histidine tag and a TEV recognition site fused at the N-terminus in plasmid pAK01. BL21-DE3 cells transformed with pAK01 were grown at 37° to an OD of 0.6 and induced for 4 h with 0.1 mM IPTG, harvested by centrifugation, suspended in 40 mL binding buffer (20 mM Tris-Cl pH 8.0, 0.5 M NaCl, 5 mM imidazole), and frozen at −80 °C. After thawing, NP40 was added to 0.1% and cells were lysed by sonication. The lysate was cleared by centrifugation (11,000× *g*, 30 min, 4 °C) and the supernatant was mixed with 4 mL of a 1:1 suspension of Ni-NTA beads (Qiagen), and then rotated at 4 °C for 1 h. The beads were washed twice with 25 mL binding buffer, suspended in 10 mL of binding buffer, then poured into a disposable column. The beads were washed with 8 mL of wash buffer (20 mM Tris-Cl pH 8.0, 0.5 M NaCl, 15 mM imidazole), followed by 6 × 1 mL aliquots of elution buffer (20 mM Tris-Cl pH 8.0, 0.5 M NaCl, 100 mM imidazole). Fractions with Hmo1 were concentrated and loaded to a Sephacryl S300 column equilibrated with 20 mM Tris-Cl pH 7.5, 0.2 M NaCl, 10% *w/v* glycerol and fractions with Hmo1 were concentrated, frozen in liquid nitrogen, and stored at −80 °C.

Primers: pTF1608 5′-CCGGCATATGACTACAGATCCTTCTGTCAAATTGAAG
5′-GGCCGGATCCGTAATAGTAACGAGTTTGTCCGTCC

Recombinant linker histone H1.0 from *Xenopus laevis* was expressed in *Escherichia coli* using described protocol [43].

### 2.3. Nucleosomal DNA Templates

**Figure 1:**
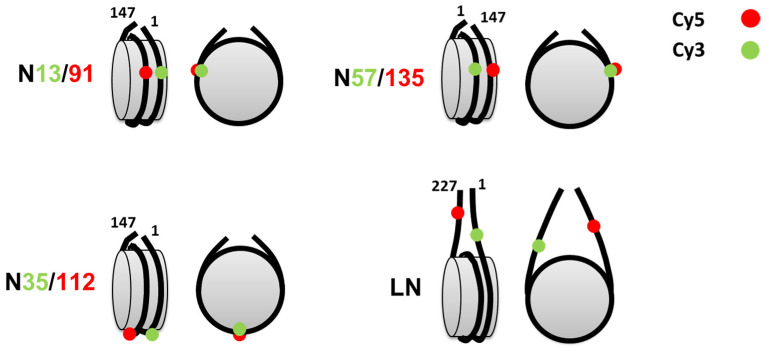
Mononucleosomes used for the analysis of Hmo1, FACT, and H1-binding. Each core (N13/91, N35/112, N57/135) and linker DNA-containing (LN) nucleosome contained a single pair of labels Cy3 and Cy5 (green and red circles, respectively) attached to DNA at the indicated positions (distances from the nucleosome boundary).

Core nucleosomes (nucleosomes without linker DNA) N13/91, N35/112, N57/135, and nucleosomes LN having linker DNA (Figure 1) were assembled using corresponding nucleosomal DNA templates containing fluorescent labels Cy3 and Cy5. DNA templates for N13/91, N35/112, and N57/135 nucleosomes were obtained by polymerase chain reaction (PCR) using the fluorescently labeled primers (Lumiprobe, Russia) and a plasmid containing the modified Widom 603–42 sequence [44].

The nucleosome positioning s603–42 sequence was

5′-CCGGGATCCAG
ATCCCGAAAATTTATCAAAAAGAGTATTGACTTAAAGTCTAACCTATAGGATACTTACAGCCATCGAGAGGGACACGGCGAAAAGCCAACCCAAGCGACACCGGCACTGGGCCCGGTTCGCGCTCCCGCCTTCCGTGTGTTGTCGTCTCTCGGGCGTCTAAGTACGCTTAGCGCACGGTAGAGCGCAATCCAAGGCTAACCACCGTGCATCGATGTTGAAAGAGGCCCTCCGTCCTTATTACTTCAAGTCCCTGGGGT-3′.

Forward and reverse primers for the DNA template of N13/91 were

5′-CCCGGTTCGCGC[Cy3-dT]CCCGCCTTCCGTGTGTTGTCGTCTCTCGG-3′ and
5′-ACCCCAGGGACTTGAAGTAATAAGGACGGAGGGCCTCTTTCAACATCGATGCACGG[Cy5-dT]GGTTAG-3′, respectively.

Forward and reverse primers for the DNA template of N35/112 were

5′-CCCGGTTCGCGCTCCCGCCTTCCGTGTGTTGTCG[Cy5-dT]CTCTCGG-3′ and
5′-ACCCCAGGGACTTGAAGTAATAAGGACGGAGGGCC[Cy3-dT]CTTTCAACATCGAT-3′, respectively.

Forward and reverse primers for the DNA template of N57/135 were

5′-CCCGGTTCGCGCTCCCGCCTTCCGTGTGTTGTCGTCTCTCGGGCGTCTAAGTACGC[Cy3-dT]TAGGC-3′ and
5′-ACCCCAGGGACT[Cy5-dT]GAAGTAATAAGGACGGAGGGCCTCTTTC-3′, respectively.

DNA template for LN nucleosomes was obtained in two-step PCR as described earlier [45]. In the first step the primers were used that were complementary to the terminal fragments of the 603 Widom sequence [46]:

5′-CACCGGCACGAGGGCCCGGTTC-3′ (forward primer) and
5′-ACTTTCTGGCAAGAAAATGAGCT-3′ (reverse primer).

The resultant DNA (180 bp) was used as the template in the second PCR with the fluorescently labeled oligonucleotides (Syntol, Russia):

5′-TAAGGCGAATTCACAACTTTTTGGC[Cy5-dT]AGAAAATGAGCT-3′ (forward primer) and
5′-ACACGGCGCACTGCCAACCCAAACGACACC[Cy3-dT]GCACGAG-3′ (reverse primer).

The DNA templates were purified with the Evrogen Purification Kit (Evrogen, Russia) and analyzed in native electrophoresis in 4% polyacrylamide gel (PAAG) in 0.5 × TBE buffer (45 mM Tris, 45 mM borate,1 mM EDTA), detection was performed with Amersham Typhoon RGB imager (Cytiva, Sweden) in the Cy3 and Cy5 specific ranges of excitation and emission. 

### 2.4. Nucleosome Assembly and Purification

Nucleosomes were formed by the transfer of histone octamers from the H1-free chromatin of chicken erythrocytes to the corresponding DNA template using stepwise salt dialysis, as described previously [47]. Nucleosomes were purified from an excess of donor chromatin and nonspecific products as described previously [13]. The efficiency of nucleosome assembly and purification was monitored with an Amersham Typhoon RGB imager after native electrophoresis in 4% PAAG. Nucleosomes were detected using excitation at 532 nm and emission at 580 nm (Cy3 signal) or 670 nm (Cy3-to-Cy5 FRET). 

### 2.5. EMSA Analysis

The electrophoretic mobility shift assay (EMSA) was performed in a native 4% PAAG in 0.5 × TBE buffer (45 mM Tris, 45 mM borate, 1 mM EDTA) at 120 V for 30–40 min or in 0.4 × TBE at 80 V for 60 min. Nucleosomes and protein-nucleosome complexes were detected in gels using the Amersham Typhoon RGB imager as described above or with a Bio-Rad ChemiDoc MP imager. Hmo1, Nhp6, and FACT (Spt16-Pob3) at the concentrations indicated in each experiment were added to nucleosomes, incubated for 10 min at 30 °C in buffer A (17 mM HEPES pH 7.6, 2 mM Tris-HCl, 0.8 mM Na3EDTA, 0.11 mM 2-mercaptoethanol, 11 mM NaCl, 1.1% glycerol and 12% sucrose). If used, competitor DNA (10 mg/mL, salmon sperm, Sigma, USA) was added to the samples immediately before electrophoresis.

### 2.6. spFRET Experiments

Single-particle FRET (spFRET) measurements were conducted with an LSM710-ConfoCor3 confocal microscope (Carl Zeiss, Germany) as described previously [13]. Nucleosomes N13/91, N35/112, and N57/135 in buffer A were used at 1 nM, with Hmo1 (1.33 μM), Nhp6 (1.33 μM), and FACT (0.13 μM) added as indicated in each experiment, incubated for 10 min at 37 °C, then subjected to spFRET measurements in a multiwell silicon chamber (Ibidi GmbH, Germany) fixed on a cover glass. Pre-formed protein-nucleosome complexes were mixed with competitor DNA (10 mg/mL, salmon sperm, Sigma, USA) directly on the microscope stage, followed by spFRET measurements.

LN nucleosomes (1 nM) were studied in a buffer B containing 10 mM Tris-HCl (pH 8.0), 1 mM 2-mercaptoethanol, 0.5 mM Na_3_EDTA, and 150 mM KCl. Hmo1 (0.33 μM or 1.33 μM) was mixed with nucleosomes (1 nM) incubated for 10 min at 37 °C and subjected to spFRET as described above. For chromatosome formation, H1.0 (100 nM) was added to LN in buffer B and incubated for 10 min at 30 °C. Half of the sample was measured, while the other half of the sample was further incubated with Hmo1 (1.33 μM) for 10 min at 30 °C prior to assaying.

In the highly diluted solution (conditions used here), single nucleosomes or their complexes with proteins randomly diffused through the focus of a laser beam (where the intensities of fluorescence of Cy3 and Cy5 labels attached to the DNA) were measured and used to estimate the efficiency of FRET between the labels [48]. Therefore, FRET efficiency reports on the distance between the Cy3 and Cy5 labels attached to the DNA and can detect changes in the proximity of the labeled sites in the range of ~4 to ~9 nm. Donor-acceptor pairs were placed in different regions of nucleosomes to probe local changes in the distances between DNA sites upon binding of factors to the nucleosomes. Additional details of FRET and spFRET techniques can be found elsewhere [49]. 

The spFRET measurements were carried out over a time course of 10–15 min. The results of the measurements were presented as relative frequency distributions of nucleosomes by the proximity ratio E_PR_ (E_PR_ profiles) as described [48]. E_PR_ is an analog of FRET efficiency without correction for fluorescence quantum yields of labels and differences in detection sensitivity. Sample sizes varied from 3000 to 8000 nucleosomes per measurement. E_PR_ profiles were further approximated as a superposition of one, two, or three Gaussian curves, where each Gaussian corresponded to a particular subpopulation of nucleosomes with different E_PR_ values. The content of each nucleosome subpopulation was calculated as the ratio of the area under the corresponding Gaussian peak to the area under the entire E_PR_ profile. The E_PR_ profiles and contents of nucleosome subpopulations were averaged (mean ± SEM) over three independent experiments. Statistical significance of differences in the analyzed data was analyzed with the two-tailed unpaired t-test.

### 2.7. Genetic Analysis

Cultures of strains with the genotypes shown were constructed using standard genetic crosses and grown in rich medium to saturation. 10-fold serial dilutions were prepared in sterile water and aliquots were spotted to the media indicated. Incubation times and temperatures are indicated in each experiment.

## 3. Results

### 3.1. Studying Mononucleosomes by spFRET

Interactions of Hmo1 with nucleosomes were studied using four types of fluorescently labeled mononucleosomes (Figure 1): core nucleosomes N13/91, N35/112, and N57/135 that were assembled on 147 bp DNA templates based on the Widom s603 nucleosome positioning sequence and a nucleosome with linkers (LN) that was assembled on 227 a bp DNA template containing the s603 sequence flanked by two 40-bp linker DNA fragments. Each nucleosomal DNA N13/91, N35/112, and N57/135 contained a pair of fluorescent Cy3 and Cy5 labels attached to thymine bases at positions 13 and 91 bp, 35 and 112 bp, or 57 and 135 bp from the beginning of the s603 sequence, respectively. Labels were placed on bases that face outward in assembled nucleosomes to avoid disturbing DNA-histone contacts that contribute to nucleosome assembly or stability. Locations were chosen to optimize the proximity of the label pairs to allow FRET efficiency to be used to measure changes in the distance between gyres within nucleosomes or between linkers adjacent to nucleosomes [49,50] (Figure 1). Positions near the nucleosome boundaries (N13/91 and N57/135) and where H2A/H2B dimers contact DNA (N35/112) were chosen to probe these distinct environments within nucleosomes (Figure 1). The selection of these label positions provided a direct comparison of the effects of Hmo1 with those revealed previously for Nhp6 using the same strategy of nucleosome labeling [51]. LN nucleosomes contained fluorescent labels in the DNA linkers 10 bp before and 15 bp after the entry/exit sites of the s603 DNA sequence (Figure 1) that allowed monitoring of conformational changes in the linkers adjacent to the nucleosomes upon chromatosome formation [45,50].

Structural changes in nucleosomes induced by Hmo1, FACT, and histone H1 were monitored using spFRET or in-gel FRET imaging. These techniques detect changes in the distance between the labeled DNA sites that alter the efficiency of FRET between the fluorophores—reporters. The formation of complexes was monitored with EMSA.

### 3.2. Hmo1 Affected Nucleosome Structure

**Figure 2:**
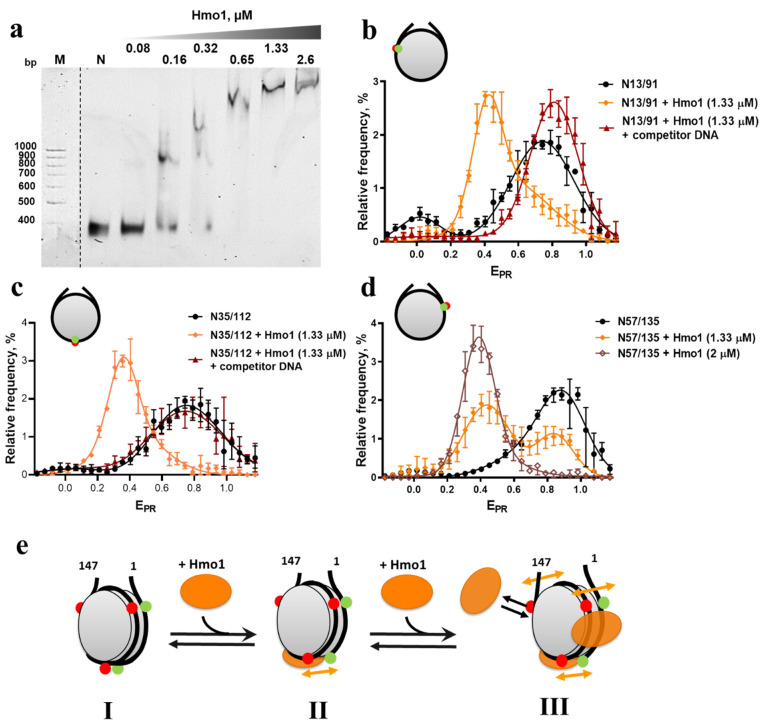
Analysis of interactions of Hmo1 with fluorescently labeled core nucleosomes. (**a**) EMSA of core nucleosomes (N57/135) and their complexes with Hmo1 (N:Hmo1). M—DNA markers. N—nucleosomes. (**b**–**d**) spFRET analysis of Hmo1 interactions with N13/91 (**b**), N35/112 (**c**), and N57/135 (**d**). Frequency distributions of fluorescently labeled nucleosomes by E_PR_ are shown for free nucleosomes (~1 nM) and nucleosome complexes with Hmo1 before and after the addition of competitor DNA (10 mg/mL). Competitor DNA itself does not affect nucleosome structure (Figure S2). The maxima of E_PR_ peaks (mean ± SEM, n = 3) and numbers of single particles observed (N) were: N13/91 (0.02 ± 0.01, 0.75 ± 0.01; N = 3218); N13/91 + Hmo1 (0.45+0.01; N = 2730); N13/91 + Hmo1 + competitor DNA (0.81 ± 0.01; N = 4908); N35/112 (0.01 ± 0.02, 0.75 ± 0.02; N = 3146); N35/112 + Hmo1 (0.38 ± 0.00; N = 1553); N35/112 + Hmo1 + competitor DNA (0.01 ± 0.02, 0.75 ± 0.02; N = 11257); N57/135 (0.00 ± 0.01, 0.86 ± 0.01; N = 5651); N57/135 + Hmo1 (1.33 μM) (0.02 ± 0.01, 0.44 ± 0.01, 0.85 ± 0.01; N = 5852); N57/135 + Hmo1 (2 μM) (0.39 ± 0.01; N = 1018). The E_PR_ profiles were averaged over three independent experiments and fitted with two Gaussian distributions except for N57/135 + Hmo1 (1.33 μM), which was fitted to three Gaussian distributions. (**e**) Schematic model of structure changes in nucleosomal DNA caused by Hmo1 and the proposed positions of Hmo1 binding sites on the nucleosome. I—intact nucleosome. II—Hmo1 binds to nucleosomal DNA and primarily affects its conformation in the area of contact with H2A/H2B dimers. III—Further binding of Hmo1 causes changes in the conformation of DNA near the boundaries of the nucleosome. Red and green dots mark positions of Cy3 and Cy5 labels, respectively. Arrows indicate an increase in the distance between DNA gyres caused by Hmo1 binding. The structural changes caused by Hmo1 in the nucleosome are reversible.

Hmo1 altered the mobility of nucleosomes during non-denaturing PAAG electrophoresis (PAGE), suggesting the formation of complexes (Figure 2a and Figure S1). Core nucleosomes formed complexes at concentrations of Hmo1 of 166 nM, with higher concentrations leading to the formation of slower-migrating forms suggesting multiple binding sites within core nucleosomes (Figure 2a). Titration of Hmo1 with nucleosomes with short linkers (181 bp 5S RNA, supplemental methods, Figure S1) also produced three or more distinct migration forms, consistent with multiple binding sites. Previously published results indicated that multiple Hmo1 molecules can bind a single DNA molecule [32,52,53] and that Hmo1 can form higher order oligomers in solution [52,53], potentially explaining the continued retardation of migration at higher Hmo1 concentrations (Figure 2a and Figure S1). However, since structural changes in different parts of the nucleosome occur at different concentrations of Hmo1 (see text below, Figure 2b–d), the presence of several binding sites for Hmo1 on a nucleosome (including some in the nucleosomal core) is very likely.

spFRET analysis revealed high E_PR_ values for N13/91, N35/112, and N57/135 bp (Figure 2b–d), confirming that the positions of the Cy3/Cy5 labels in these DNAs were brought into a close enough proximity to produce efficient FRET in these nucleosomes. A small subpopulation of N13/91 (14%) displayed lower E_PR_ values near zero, consistent with “breathing” or spontaneous temporary unwinding of the DNA near the boundary of a nucleosome [54].

The addition of Hmo1 caused a significant reduction in the E_PR_ values in all cases (Figure 2b–d), indicating increased distances between the gyres at all three locations within the nucleosomes. This indicates that the conformation of the DNA in nucleosomes was globally altered in the complexes with Hmo1 that were detected by EMSA. The shift in E_PR_ was concentration-dependent (Figure 2d) and the effect was reversible (lost upon addition of an excess of competitor DNA; Figure 2b,c and Figure S1). This is consistent with the dynamic binding of Hmo1 at multiple sites, with the position marked in N57/135 being most resistant to the effects of Hmo1 (least shifted at 1.33 µM Hmo1), possibly revealing a hierarchy of occupancy among the sites. Notably, the E_PR_ values were reduced to a similar level in each case, but not to zero, indicating that the reporter dye positions remained in close proximity while being displaced further from one another than they were in the free nucleosomes. This suggests that Hmo1 bound to the nucleosome and increased the separation of the DNA gyres throughout the core, but did not fully unwind the DNA (Figure 2e).

### 3.3. Effect of Hmo1 on Linker DNA in Nucleosomes and Chromatosomes

**Figure 3:**
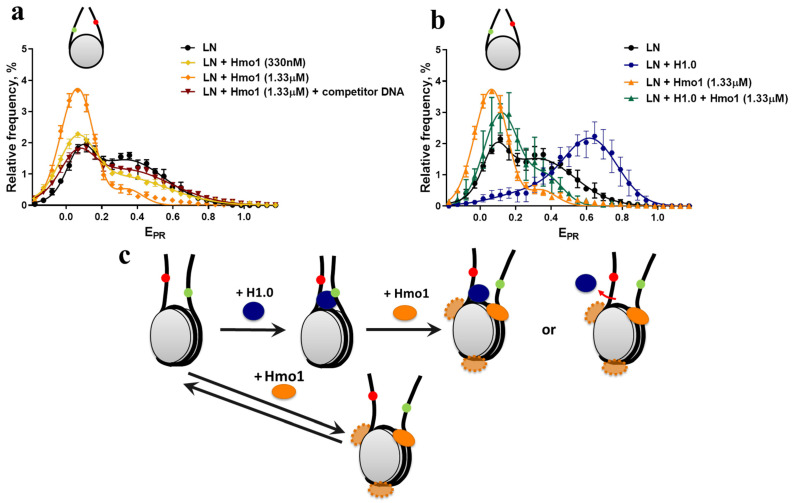
spFRET analysis of the effect of Hmo1 on the configuration of linker DNA regions of nucleosomes and chromatosomes. (**a**) Frequency distributions of nucleosomes by E_PR_ values are shown for free LN nucleosomes (~1 nM), their complexes with Hmo1 at concentrations of 330 nM and 1.33 µM, and complexes of LN with Hmo1 after the addition of competitor DNA (0.5 μM). The maxima of E_PR_ peaks (mean ± SEM, n = 3) and the numbers of single particles observed (N) were: LN (0.09 ± 0.01, 0.37 ± 0.01; N = 4595); LN + 0.33 μM Hmo1 (0.07 ± 0.01, 0.36 ± 0.01; N = 13762); LN + 1.33 μM Hmo1 (0.06 ± 0.01; N = 6649); LN + Hmo1 + competitor DNA (0.07 ± 0.01, 0.37 ± 0.02 N = 8744). (**b**) Frequency distributions of nucleosomes by E_PR_ values are shown for free LN nucleosomes (~1 nM), their complexes with H1 (100 nM) (chromatosomes), LN complexes with Hmo1 (1.33 µM), chromatosomes + Hmo1 (1.33 µM). The maxima of E_PR_ peaks (mean ± SEM, n = 3) and the numbers of single particles observed (N) were: LN (0.09 ± 0.00, 0.37 ± 0.01; N = 4595); LN + Hmo1 (0.06 ± 0.00; N = 6649); LN + H1.0 (0.22 ± 0.03, 0.62 ± 0.04; N = 11226); LN + H1.0 + Hmo1 (0.20 ± 0.05, 0.40 ± 0.02; N = 1413). The E_PR_ profiles (**b**,**c**) were averaged over three independent experiments and fitted with two Gaussian curves. (**c**) A schematic model of the conformational changes in the linker DNA region of nucleosomes and chromatosomes caused by Hmo1. Red and green dots mark positions of Cy3 and Cy5 labels, respectively. Several molecules of Hmo1 bind to a nucleosome and a chromatosome and cause an increase in the distance between DNA linkers, and, as shown in Figure 2, in the distance between DNA gyres in the core region. H1 and Hmo1 are proposed to bind to independent sites, so H1 can remain bound in the presence of bound Hmo1.

When the reporter dyes were placed in linker DNA segments, spFRET revealed at least two overlapping populations with E_PR_ maxima at 0.09 and 0.37 ([45] and Figure 3a), indicating multiple conformations of the linkers with a 2:1 preference for the form producing higher FRET. The binding of Hmo1 preferentially disrupted the high FRET form and reduced the E_PR_ value of the remaining nucleosomes slightly to a peak at 0.06 (Figure 3a). The effect of Hmo1 was reversed by addition of competitor DNA, indicating that Hmo1 binding was dynamic and caused the separation of the linkers from one another, perhaps by bending the DNA at the entry/exit sites as proposed in Figure 3c.

In contrast, the addition of the linker histone H1.0 caused a shift toward higher E_PR_ values with a peak at 0.62, demonstrating that the linkers moved closer together (Figure 3b) as expected for the formation of the more compact chromatosome form [45]. These opposing effects of Hmo1 and H1.0 do not support the earlier proposal that Hmo1 has properties similar to linker histones [37,38]. 

Combining Hmo1 and H1.0 with nucleosomes resulted in predominantly one population with an E_PR_ maximum at 0.11 ± 0.01, while Hmo1-LN complexes had a peak at 0.058 ± 0.006 (mean ± SEM, n = 3; Figure 3b). The difference between the Hmo1-LN populations with and without H1.0 was statistically significant (*p* = 0.015), suggesting that H1 remained associated with the Hmo1-LN complexes, but was unable to maintain the linkers in the chromatosomal configuration (Figure 3c). We propose that Hmo1 and H1 bind to distinct sites, and that Hmo1 acts as an architectural protein that promotes linker DNA separation rather than the compaction expected for a linker histone.

### 3.4. Hmo1 Facilitates Unwrapping of Nucleosomal DNA by FACT

**Figure 4:**
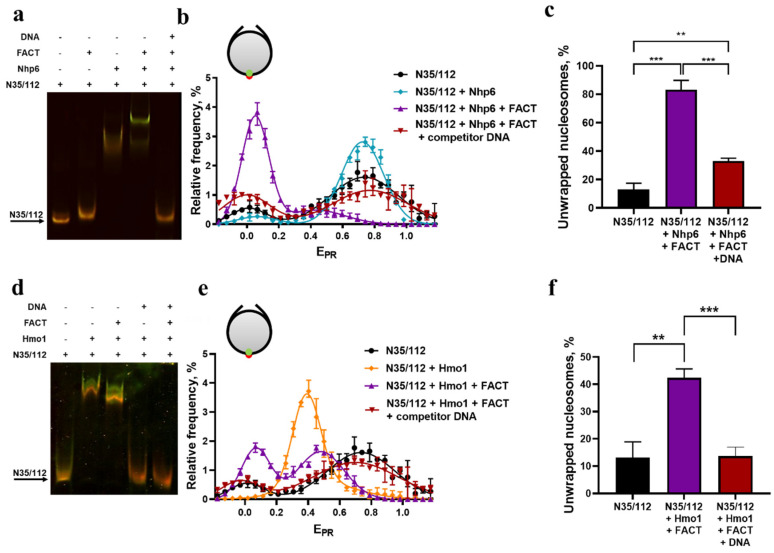
Analysis of FACT interactions with nucleosomes in the presence of Nhp6 or Hmo1. (**a**,**d**) EMSA and in-gel FRET analysis of interactions between nucleosomes N35/112 (~1 nM), FACT (0.133 μM), Nhp6 (1.33 μM) or Hmo1 (1.33 μM) and competitor DNA (0.5 μM). An orange-yellow color indicates a higher FRET between Cy3 and Cy5 in nucleosomes while green indicates lower FRET. **(b**,**e**) spFRET analysis of FACT-induced reorganization of nucleosomes in the presence of Nhp6 (**b**) or Hmo1 (**e**). Frequency distributions of N35/112 nucleosomes by E_PR_ are shown for: (**b**) free nucleosomes (~1 nM), N35/112 complexes with Nhp6 (1.33 μM), with Nhp6 (1.33 μM) and FACT (0.133 μM) or with Nhp6 (1.33 μM) and FACT (0.133 μM) and competitor DNA (0.5 μM). (**e**) As in b but with free nucleosomes (~1 nM) and N35/112 complexes with Hmo1 (1.33 μM), with Hmo1 (1.33 μM) and FACT (0.33 μM) or with Hmo1 (1.33 μM), FACT (0.133 μM) and competitor non-labeled DNA (0.5 μM). In agreement with the data published previously [51] FACT itself does not interact with a nucleosome (Figure S3). Competitor DNA does not affect nucleosome structure (Figure S2). The maxima of E_PR_ peaks (mean ± SEM, n = 3) and the numbers of single particles observed (N) were: N35/112 (0.02 ± 0.01, 0.74 ± 0.01; N = 6357); N35/112 + Nhp6 (0.06 ± 0.01, 0.74 ± 0.01; N = 5383); N35/112 + Nhp6 + FACT (0.05 ± 0.01, 0.46 ± 0.02; N = 8354); N35/112 + Nhp6 + FACT + competitor DNA (0.02 ± 0.02, 0.77 ± 0.02, N = 3811); N35/112 + Hmo1 (0.43 ± 0.01; N = 1018); N35/112 + Hmo1 + FACT (0.07 ± 0.00, 0.48 ± 0.00; N = 19494); N35/112 + Hmo1 + FACT+ competitor DNA (0.04 ± 0.0, 0.70 ± 0.01; N = 14483).The E_PR_ profiles (**b**,**e**) were averaged over three independent experiments and fitted with two Gaussian distributions except for N35/112 + Hmo1 which was fitted to a single Gaussian distribution. (**c**,**f**) Comparison of the subpopulations of unwrapped nucleosomes according to the spFRET analysis presented in panels b and e. Each subpopulation was calculated as the area under the low FRET Gaussian curve (E_PR_ < 0.2) to the total area under the E_PR_ profile (in %). Averaged subpopulations of unwrapped nucleosomes (mean ± SEM, n = 3) were: 13 ± 3% for N35/112, 83 ± 3% for N35/112- Nhp6-FACT complexes; 33 ± 1% for the mixture of N35/112, Nhp6, FACT and competitor DNA; 42 ± 1% for N35/112-Hmo1-FACT complexes; 13 ± 1% for the mixture of N35/112, Hmo1, FACT and competitor DNA. The results of t tests are summarized as ** = *p* < 0.005, and *** = *p* < 0.0005.

Yeast FACT (Spt16-Pob3) required the addition of Nhp6 to achieve large-scale unfolding of nucleosomal DNA in the spFRET assay [22,51]. Given the high sequence homology between the dual HMGB domains of Hmo1 and the single domain of Nhp6 [7], we asked whether Hmo1 could substitute for Nhp6 for this activity. Nucleosome N35/112 was mixed with FACT, Nhp6, Hmo1, or combinations and analyzed by EMSA and spFRET (Figure 4). In this case, the EMSA gel was illuminated at the excitation wavelength of Cy3 (532 nm), then emission was detected at 580 nm (Cy3) and 670 nM (Cy5), with the latter indicating in-gel FRET (orange bands in Figure 4a,d). Robust FRET was detected for nucleosomes alone or nucleosomes bound by Nhp6, but FRET was reduced for complexes containing Hmo1, FACT with Nhp6, or FACT + Hmo1 (green or yellow-green bands in Figure 4a,d). These changes were reversed by addition of competitor DNA prior to electrophoresis.

**Figure 5:**
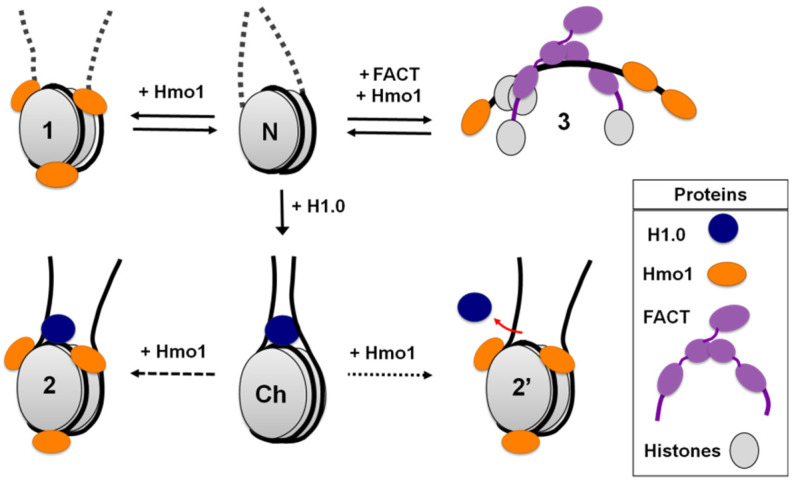
A model for the changes in nucleosome structure induced by Hmo1 alone or with FACT. Hmo1 interacts with nucleosomes (N, 1) and chromatosomes (Ch, 2, 2′) and facilitates FACT-dependent, ATP-independent nucleosome unfolding (3). Hmo1 binding to nucleosomes induces a significant increase in the distance between the gyres of core nucleosomal DNA and DNA linkers (1). Interaction of Hmo1 with chromatosomes affects their structure resulting in DNA linkers coming apart (2, 2′). As shown in Figure 3, there is a closer proximity of helices of linker DNA in chromatosomes in presence of Hmo1 than in nucleosomes, so we propose that linker histone is more likely to stay bound along with Hmo1 (2), with dissociation occurring independently (2′). Hmo1 mediates FACT binding to nucleosomes and facilitates its ATP-independent reversible nucleosome reorganization (3).

spFRET confirmed the previously described nearly complete unwrapping of nucleosomal DNA by FACT + Nhp6 [15] (Figure 4b,c). Importantly, the partial unwrapping induced by Hmo1 alone (Figure 2c and Figure 5) was enhanced by the addition of FACT to produce a new population with an E_PR_ of 0.05 ± 0.01 (Figure 4e,f). The conversion to this form was less efficient with Hmo1 than with Nhp6 (Figure 4c,f; 42.4 ± 1.3% vs. 83 ± 3% or more [51], with similar results using different ratios of Hmo1 and FACT as shown in Figure S4). However, the nearly complete loss of FRET between reporters near the center of the nucleosomal DNA fragment suggests that Hmo1 + FACT produced large-scale unwrapping to a nearly linear structure (Figure 5) similar to the one produced by Nhp6 + FACT [15].

### 3.5. Hmo1 Supports FACT Function In Vivo

**Figure 6:**
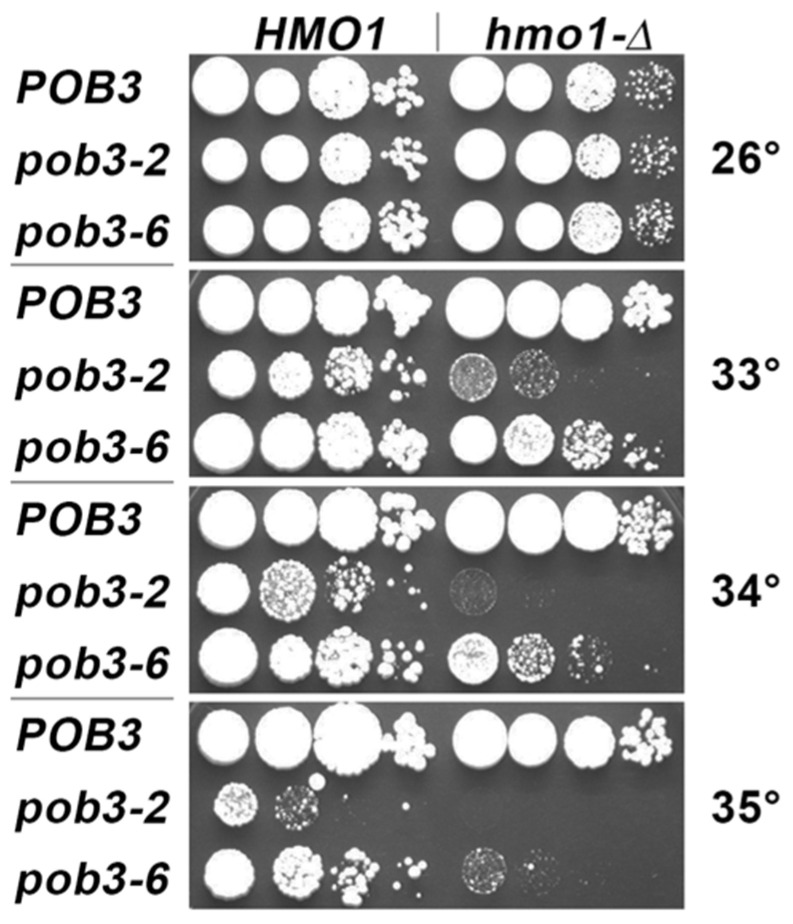
Combining a deletion of *HMO1* with unstable hypomorphs of *POB3* resulted in synthetic growth defects, detected as enhanced temperature sensitivity. Strain 7697 (HMO1) and 7831-1-3 (hmo1-∆) carrying derivatives of pTF139 with the alleles of POB3 indicated [56] were incubated at the temperatures indicated for three days. Deletion of *HMO1* caused a growth delay (also see Figure S5). This defect was not altered by pob3-2 or pob3-6 at the permissive temperature of 26 °C, but synthetic growth defects were observed at higher temperatures.

While Spt16 and Pob3 are both essential for the viability of *S. cerevisiae*, hypomorphs with reduced activity have been isolated and display severe synthetic defects with loss of Nhp6, indicating a role for Nhp6 is supporting FACT activity in vivo [22]. Similarly, loss of Hmo1 caused enhanced temperature sensitivity caused by alleles of Spt16 and Pob3 that reduce the stability of these proteins ([55]; Figure 6 and Figure S5a). Loss of Hmo1 also caused sensitivity to the DNA damaging agent phleomycin, and this was exacerbated by other alleles of Spt16 and Pob3 that produce stable proteins with functional defects, whereas combining these mutations lead to some suppression of sensitivity to hydroxyurea and caffeine (Figure S5b). These genetic interactions indicate that FACT and Hmo1 work together in vivo in distinct ways for different functions, suggesting complex, pathway-specific roles for Hmo1 and Nhp6.

## 4. Discussion

Taken together, our data revealed several novel functions of Hmo1: it interacted with and changed the conformation of core nucleosomes (Figure 2, Figure 3, Figure 5, and Figure S1) and chromatosomes (Figure 3 and Figure 5), and it supported the binding of the histone chaperone FACT to nucleosomes, and it also supported large-scale ATP-independent, reversible nucleosome unfolding by FACT (Figure 4 and Figure 5). Finally, loss of Hmo1 enhanced some phenotypes caused by hypomorphic alleles of FACT, suggesting they collaborate functionally in vivo (Figure 6 and Figure S5).

Hmo1 is a DNA-binding protein that induces the bending of linear DNA [32,57]. It has been proposed that Hmo1 would bind exclusively to linker DNA adjacent to nucleosomes [36], but we found that even core nucleosomes lacking linkers appear to bind several molecules of Hmo1 simultaneously. The binding site for Hmo1 in free DNA has been estimated to be about 26 bp [32]. Binding of one Hmo1 monomer to ~26 bp of nucleosomal DNA would occlude a similar amount of the adjacent nucleosomal DNA gyre, so binding of three monomers would saturate the available DNA in a nucleosome. This tight positioning of three Hmo1 molecules on the nucleosome surface could be facilitated by the ability of Hmo1 to form homo-multimeric complexes [52,53].

We observed increases in the distance between gyres upon binding of Hmo1 at all three sites tested, including the 35/112 position furthest from the entry/exit points that is in contact with the H2A-H2B histone dimers. Separation of the DNA gyres could modulate the accessibility of surfaces that are occluded in canonical nucleosomes, enhancing binding by other factors like FACT that have an affinity for these surfaces, or it could promote disruption of histone-DNA contacts that are barriers to remodeling, transcription, or other processes. The effects of Hmo1 on nucleosomes were uniformly reversible by the addition of competitor DNA, demonstrating the dynamic nature of these changes.

Hmo1 also induced an increase in the distance between the helices of linker DNA (Figure 3a,b). This effect is opposite to the activity of histone H1, which brings the helices of linker DNA closer together (Figure 3b). The observed effect of Hmo1 is similar to the effects of other HMGB proteins on linker DNA, which generally increase nucleosomal DNA accessibility “leaving the gate ajar for transcription to occur” [58]. In agreement with this conclusion, Hmo1 still increased the separation of the linker segments in chromatosomes formed with H1.0 (Figure 3b and Figure 5). Therefore, our data favor the interpretation that Hmo1 is a typical architectural factor like other HMGB proteins [28], not a linker histone-like protein [37,38].

At the same time, our experiments revealed some differences between Hmo1 and some other HMGB proteins. For example, the interaction of mammalian HMGB1 with H1 occurs via the acidic terminal region of HMGB1 binding to the basic C-terminus of H1, and this interaction facilitates displacement of H1 [59,60]. Our data suggest instead that H1.0 remains bound to a nucleosome in the complex with Hmo1 (Figure 3c). Since Hmo1, like H1.0, has a basic C-terminus, we propose that Hmo1 affects the structure of the linker DNA in chromatosomes, but does not displace H1.0 from the complex.

Hmo1 was able to support FACT activity in vitro and in vivo, but it was less effective than Nhp6 at promoting complete nucleosomal DNA unwrapping (Figure 4), and the effects induced by deletion of *HMO1* (Figure 6 and Figure S5) and *Nhp6* [22] on *S. cerevisiae* were different. Our results suggest that Hmo1 might support a distinct set of FACT functions from those assisted by Nhp6, but cells with simultaneous deletion of the *nhp6a, nhp6b, hmo1,* and *nhp10* genes are still viable [31], so other factors presumably also contribute to FACT activity in yeast. 

In summary, the interaction of Hmo1 with nucleosomes caused significant changes in the conformation of the DNA throughout the core of a nucleosome or a chromatosome. This separation of the DNA gyres could promote accessibility of transcription factors, ATP-dependent remodeling complexes, and other proteins to DNA. Hmo1 also facilitated FACT-dependent nucleosome unfolding that has been proposed to increase the accessibility of nucleosomal DNA to various DNA-interacting factors and to facilitate nucleosome disruption in vivo [20,41,61,62], and this collaboration with FACT appears to be physiologically relevant.

## Data Availability

The data presented in this study are available upon request from the corresponding authors. The data are not publicly available due to local regulations.

## Supplementary Materials

The following supporting information can be downloaded at: https://www.mdpi.com/article/10.3390/cells11192931/s1, Figure S1: Electrophoresis of Hmo1 complexes with nucleosomes, which were constructed with a 181 bp DNA fragment labeled with Cy5 (green) based on the nucleosome positioning sequence from sea urchin 5S rDNA and yeast histones labeled with Oregon Green on H2A-Q114C; Figure S2: Competitor DNA itself does not affect nucleosome structure; Figure S3: FACT itself does not interact with a nucleosome; Figure S4: spFRET analysis of FACT interactions with N35/11 nucleosomes (~1 nM) in the presence of Hmo1 at different Hmo1:FACT ratios; Figure S5: Deletion of *HMO1* causes synthetic effects when combined with defective alleles of the FACT subunits. References [63,64,65] are cited in Supplementary Materials.
