# Supplementary material for: Hmo1 Protein Affects the Nucleosome Structure and Supports the Nucleosome Reorganization Activity of Yeast FACT
Source: Cells. 2022 Sep 20;11(19):2931. doi: 10.3390/cells11192931 (PMC9564320; doi:10.3390/cells11192931)
Supplement: Supplementary file 1 [file cells-11-02931-s001.zip › cells-1894196-supplementary.pdf]

Supplementary materials to

Hmo1 Protein Affects Nucleosome Structure and Supports the Nucleosome Reorganization Activity of Yeast FACT

Daria K. Malinina[1], Anastasiia L. Sivkina[1], Anna N. Korovina[1], Laura L. McCullough[2], Tim Formosa[2], Mikhail P. Kirpichnikov[1,4], Vasily M. Studitsky[1,3]*, Alexey V. Feofanov[1,4]*

1 Biology Faculty, Lomonosov Moscow State University, 119992 Moscow, Russia

2 Department of Biochemistry, University of Utah School of Medicine, Salt Lake City, Utah 84132, USA

3 Fox Chase Cancer Center, Philadelphia, PA 19111, USA

4 Shemyakin-Ovchinnikov Institute of Bioorganic Chemistry, Russian Academy of Sciences, Moscow 117997, Russia

* - corresponding authors.

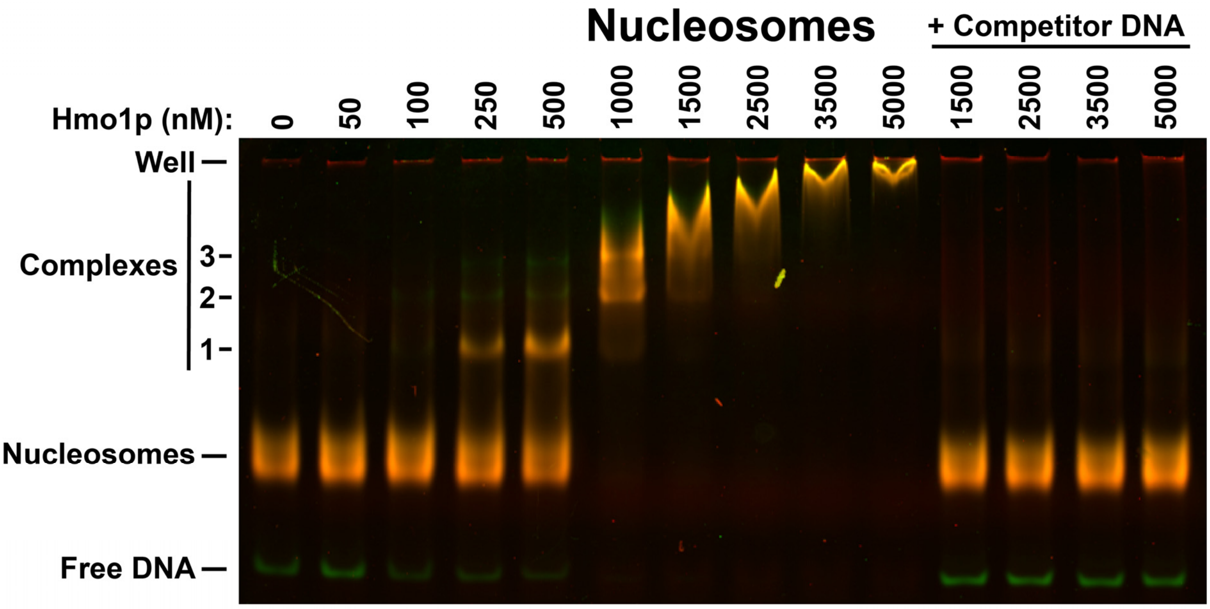

Figure S1. Nucleosomes were constructed with a 181 bp DNA fragment labeled with Cy5 (green) based on the nucleosome positioning sequence from sea urchin 5S rDNA and yeast histones labeled with Oregon Green (red in this image) on H2A-Q114C ([63]). Titrating Hmo1p with nucleosomes produced at least 3 distinct complexes with different migration rates (labeled as complexes 1, 2, 3), suggesting the potential for simultaneous occupancy of at least 3 binding sites. Addition of competitor DNA to the complexes resulted in nearly normal nucleosomes, indicating that binding of Hmo1p was largely reversible.

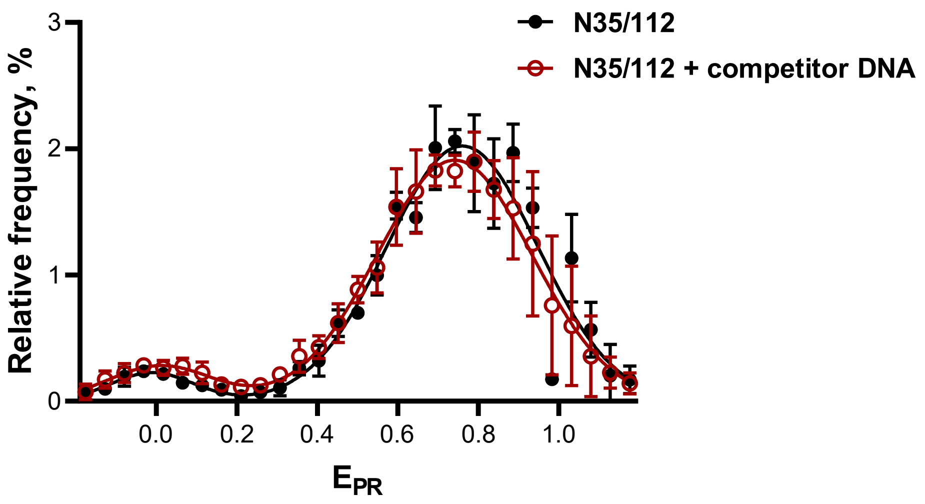

Figure S2. Competitor DNA itself does not affect nucleosome structure. Addition of competitor DNA to nucleosomes does not change the frequency distributions of fluorescently labeled nucleosomes by E_{PR}. Concentrations of nucleosomes and DNA were 1 nM and 10 mg/ml, respectively.

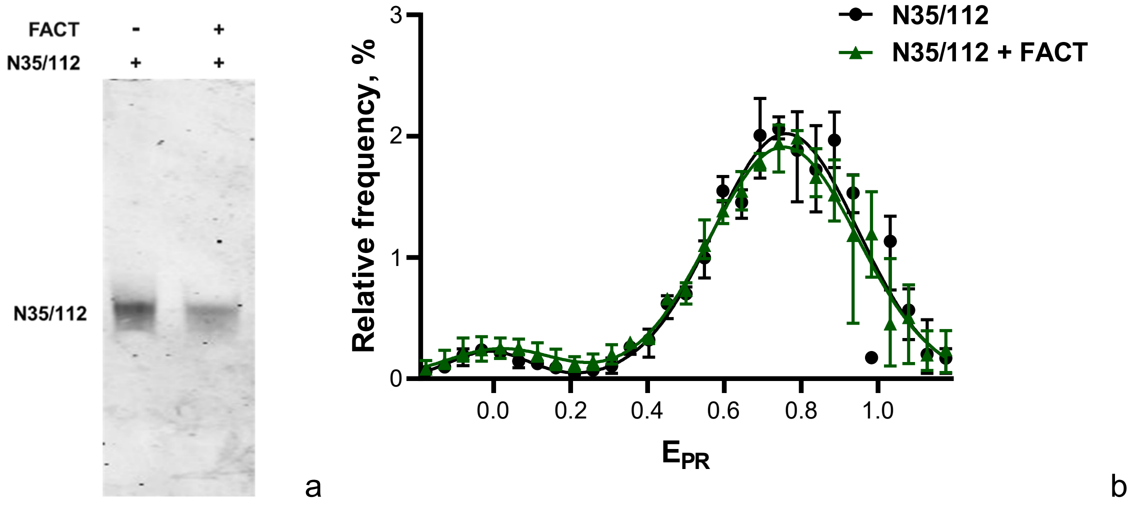

Figure S3. FACT itself does not interact with a nucleosome. Addition of FACT to nucleosomes does not lead to a shift of nucleosomes in native gel electrophoresis (a) and does not affect the structure of nucleosomes according to spFRET analysis (b). Concentrations of nucleosomes and FACT were 1 nM and 0.13 μM, respectively.

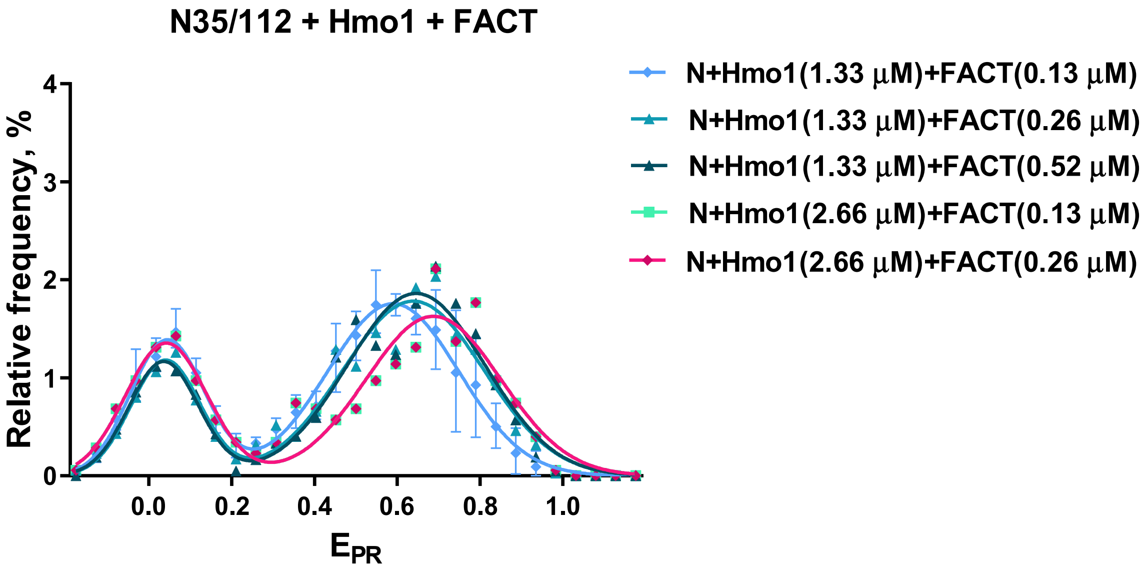

Figure S4. spFRET analysis of FACT interactions with N35/11 nucleosomes (~1 nM) in the presence of Hmo1 at different Hmo1:FACT ratios fitted with two Gaussian distributions. The efficiency of FACT-induced reorganization of nucleosomes is persistent for all used concentration ratios of Hmo1 and FACT.

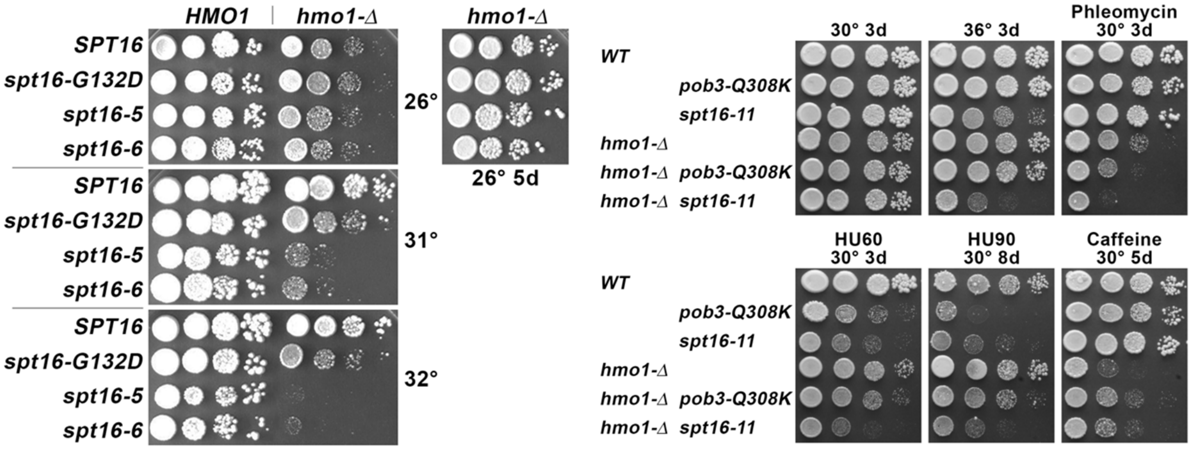

Figure S5. Deletion of *HMO1* causes synthetic effects when combined with defective alleles of the FACT subunits.

a. As in Fig 5, synthetic defects were also observed upon loss of Hmo1 expression in mutants with alleles of SPT16 that produce unstable proteins. Strain 7737-3-2 (HMO1) and 7832-1-3 (hmo1-Δ) carrying derivatives of pTF128 with the alleles of SPT16 indicated [22] were incubated at the temperatures indicated for three days. The hmo1-Δ allele caused slow growth; a picture taken after 5 days is included for just the hmo1-Δ strains at 26° demonstrating that growth was delayed not prematurely terminated.

b. Loss of Hmo1 caused a small synthetic defect in a spt16-11 strain, but not in a pob3-Q308K strain. These alleles produce thermostable proteins ([55]) that are defective in promoting or resolving reorganization of nucleosomes, respectively ([64], [65]). Loss of Hmo1 caused sensitivity to the DNA damaging agent phleomycin and the toxin caffeine, but not to the DNA replication inhibitor hydroxyurea. Genetic interactions among these mutations were complex, with enhanced defects in the presence of phleomycin, slight suppression of the defect caused by hmo1-Δ with caffeine, and

mixed effects with FACT mutations on HU (suppression of the defect caused by pob3-Q308K, slight enhancement of the defect caused by spt16-11).

Strains are from the A364a background with the following genotypes:
8127-7-4 - MATa ura3-Δ0 leu2-Δ0 trp1-Δ2 his3 lys2-128∂
8967-4-4 - MATa ura3-Δ0 leu2-Δ0 trp1-Δ2 his3 lys2-128∂ pob3-Q308K(+34, KanMX)
9495N-1-4 - MATa ura3 leu2 trp1 his3 lys2-128∂ spt16-11(+124, NatMX)
9135-4-3 - MATa ura3 leu2 trp1 his3 lys2-128∂ hmo1-Δ(::TRP1)
9623-1-4 - MATa ura3 leu2 trp1 his3 lys2-128∂ hmo1-Δ(::TRP1) pob3-Q308K(KanMX)
9623-5-2 - MATa ura3 leu2 trp1 his3 lys2-128∂ hmo1-Δ(::TRP1) spt16-11(+124, NatMX)

63.Xin, H.; Takahata, S.; Blanksma, M.; McCullough, L.; Stillman, D.J.; Formosa, T. yFACT Induces Global Accessibility ofNucleosomal DNA without H2A-H2B Displacement. *Mol. Cell* **2009**, *35*, 365–376.
64. Cells2022,11, 293116 of 1664.McCullough, L.; Rawlins, R.; Olsen, A.; Xin, H.; Stillman, D.; Formosa, T. Insight Into the Mechanism of Nucleosome Reorganiza-tion From Histone Mutants That Suppress Defects in the FACT Histone Chaperone. *Genetics* **2011**,*188*, 835–846.
65.McCullough, L.; Poe, B.; Connell, Z.; Xin, H.; Formosa, T. The FACT Histone Chaperone Guides Histone H4 Into Its NucleosomalConformation in Saccharomyces cerevisiae. *Genetics* **2013**,*195*, 101–113. [CrossRef]
